# Diagnostic imaging in the pregnant bitch: risks, advantages, and disadvantages

**DOI:** 10.3389/fvets.2025.1588847

**Published:** 2025-06-05

**Authors:** Magdalena Schrank, Maria Pereira, Antonio Mollo

**Affiliations:** Department of Animal Medicine, Production and Health, University of Padua, Padua, Italy

**Keywords:** diagnostic imaging, X-ray, ultrasound, pregnancy, dog, stress, radiation

## Abstract

Diagnostic imaging techniques are routinely used in bitches during pregnancy. Ultrasound examination (UE) and radiographic imaging (RI) are the two most frequently employed exams, for pregnancy diagnosis, assessment of fetal vitality and parturition date prediction, and for determination of litter size, respectively. In human medicine, the effects of radiation exposure resulting from diagnostic imaging, as well as radiation thresholds above which the risk of permanent damage to the offspring is significant, are well documented. Although no such information is available in veterinary medicine, breeders are increasingly skeptical about the use of X-rays in bitches in late-stage pregnancy, both because of the potential harm caused by the stress of the exam and because of the direct effects of radiation on the dam and fetuses. Stress is known to exert an effect in the reproductive processes in many species with one of the greatest stressors being the separation of the bitch from the pups for transport to the veterinary clinic during or right after parturition. This review also demonstrates that the harm resulting from radiation exposure from a radiographic study in a bitch during late-stage pregnancy and for its fetuses, who have already completed organogenesis, is poorly researched. While breeders’ hesitations may be perceived as unfounded by the veterinarians, it is crucial that they are taken into consideration and that clear communication between the breeder and veterinarian is established. Owners should be informed of the lack of studies on the subject in the domestic dog but also presented the available information for other species including the human.

## 1 Introduction

Diagnostic imaging is a critical tool in human and veterinary medicine which has been used for more than a century and undergoes continuous improvements. Effects of radiation exposure derived from diagnostic imaging are well documented, leading to the creation of strict regulations during pregnancy in the human species (1). On the other hand, the effects of radiation exposure during gestation in the bitch remain controversial with hardly any research available. Ultrasonography and radiographic imaging are the most commonly used methods of diagnostic imaging in veterinary medicine and are used routinely during gestation in the bitch. X-rays performed during the last week of gestation permit a sound estimation of litter size and its use for estimation of dystocia risk has been described. Up until today no studies have investigated the possible short and long-term effects of X-rays on embryos or fetuses in the canine species. A shift in breeders’ opinions regarding the use of radiographic imaging in the pregnant bitch has been observed. This review aims to address breeders’ and owners’ concerns about possible radiation hazards and stress effects during diagnostic imaging procedures that could impact both maternal and fetal health, evaluating the benefits and estimated risks of X-ray examinations in the latter stages of canine pregnancy.

### 1.1 Radiation

Every terrestrial organism is regularly exposed to a baseline quantity of naturally occurring environmental radiation as well as radiation derived from man-made sources (e.g., exposure to industrial, occupational and medical sources). Overall radiation may be divided in either ionizing (X-rays and gamma rays) or non-ionizing (visible light, ultraviolet, infrared, microwave, radio frequency) radiation. Background radiation exposure is mainly non-ionizing radiation (2). In the medical field, both ionizing (X-rays and gamma rays) and non-ionizing radiation (ultrasonography and magnetic resonance imaging) are used for both diagnostic and therapeutic motives (2).

Both types of radiation may have negative effects on biological tissue. These effects may be either caused by the generation of heat (non-ionizing radiation) or by directly altering the cellular structures of tissues such as proteins and DNA (2, 3).

Biological effects of radiation may be either deterministic or stochastic (2, 3). Deterministic effects derive from cellular injury, death or disintegration which present itself once the organism is exposed to a threshold dose or more (3). Contrariwise, stochastic effects describe changes on a cellular level which nevertheless permit cellular replication. No threshold dose seems to be necessary for this effect to occur, but its manifestation is dependent on both dose and time of exposure (4). To measure radiation exposure different units were introduced. Gray (Gy) is a unit used to describe the absorbed dose and is used primarily in the context of deterministic effects. Sievert (Sv) on the other hand is the dose equivalent and is used in the context of stochastic effects. Although these (Gy and Sv) units are internationally accepted SI-units, others may be found such as *rad* and *rem*. One Gy is the equivalent of 100 *rad* whereas one Sv is the equivalent of 100 *rem*. Depending on the year of publications all four units may be found in the literature. The average American citizen was estimated to be exposed to a background radiation of 0.001 Gy or 0.1 *rad* annually (5, 6).

Different types of tissue react differently to radiation exposure. High radiosensitivity is observed in the mucosa of the gastrointestinal apparatus and the haematopoetic system (7, 8). The kidney, immune system, skin and lung have also been appointed as particularly sensitive to radiation (7). The central nervous system is as well considered sensitive, especially during its embryonic development (8).

Diagnostic imaging (DI) may be considered one of the most important diagnostic tools in human and veterinary medicine. Ionizing radiation in the form of radiographic imaging (RI) (commonly referred to as X-rays) was the first diagnostic imaging technique discovered in 1895 by Wilhelm Conrad Röntgen (9). RI was introduced in veterinary medicine in 1920 (10). Investigations on the biological effects of its use started as early as 1897 (11) and increased following historical events such as the use of the atomic bombs during the second world war and the cold war between the U. S. A. and Russia (7). Interest on the negative effects of ionizing radiation was further fueled by the increased use of atomic energy in non-war related settings. RI underwent continuous improvement aimed at reducing radiation exposure while nevertheless maintaining its diagnostic utility. The discovery of the diagnostic relevance and applicability of non-ionizing radiation in the form of ultrasonography added to the possibilities of DI. In this manner, RI allowed evaluation of different anatomical structures in the form of a static image while ultrasound examination (UE) allowed a dynamic evaluation mainly used for abdominal and thoracic organs. Other uses of ionizing radiation have been developed later such as computed tomography (CT), mammography, radiation therapy and fluoroscopy. On the other hand, further investigations on the possible uses of non-ionizing radiation led to the discovery of magnetic resonance (MR) (4).

### 1.2 Radiation exposure and pregnancy

In human medicine, researchers realized quite early, that embryos and fetuses were susceptible to the negative biological effects of radiation exposure. Although protected from the outside world by many layers of tissue, embryos and fetuses are still exposed to radiation. Levels of exposure to the fetus or embryo differ from the mother’s exposure due to the protective layers. The risk for either deterministic or stochastic effects is dependent on the different developmental stages (1, 12, 13). Organogenesis is the most sensitive stage of development accounting for the period between two to 7 weeks after conception and the early fetal period between eight to 15 weeks after conception. Radiation exposure during this period may have a variety of effects which may be teratogenic, carcinogenic or mutagenic (13).

Although exposure of the human embryo/fetus is avoided as much as possible, different circumstances may result in an unforeseen exposure, such as unknown pregnancy or the necessity of diagnostic or therapeutic exposure (4). As such, it has been advised that medical professionals consider the following three principles when confronted with the necessity for diagnostic imaging in pregnant women: justification, optimization and dose limitation (5). Although much research has been performed and estimates of limits of embryonic/fetal exposure have been described, medical professionals nevertheless struggle when confronted with exposed pregnant women. Teratogenic and carcinogenic risks are perceived as particularly dangerous and high (14). Physicians may even tend to advice abortion following a diagnostic imaging procedure. This perception of risk by medical staff has been described as being excessive (15). Although limited, research in humans is much advanced when compared to veterinary species. Tresholds of pre-natal radiation exposure have been defined for potential non-cancerous health effects (failure to implant, miscarriage, malformations, growth restrictions and others) depending on the stage of pregnancy during exposure (blastogenesis, organogenesis and fetogenesis) (2, 16). Noncancer health effects are not observed at a fetal radiation exposure threshold dose below 0.05 Gy (5 rad) at any stage of gestation. Also, risk of cancer increases with prenatal exposure to radiation and intervals of in-utero radiation dosages have been correlated with estimated incidence of childhood cancer. Other specific threshold values can be consulted elsewhere (2, 16).

### 1.3 Animal studies and animal models

Animal studies and animal models have always been of great research importance in human medicine. Negative side effects of radiation exposure have been and still are investigated using experimental animal studies. Studies in the 1900s used different domestic animal species to evaluate the reaction of the organism on experimental exposure to different levels and types of radiation (17–21). With increased acknowledgement of ethical concerns regarding experiments on domestic animals, researchers started to focus either on the use of biological specimen rather than living animals (22), or on the use of mice and rats in laboratory settings (7, 23). Overall, very little is known about the canine organism concerning radiation exposure, as studies mainly focused on bovine and pigs (8, 21). The above-cited thresholds defined for the human species on the effect of radiation exposure during pregnancy on embryo and fetal development are missing in veterinary medicine. Yet, the LD_50/30_, which represents the full-body radiation dose necessary to cause death of half the irradiated animals within 30 days, is known in different species (donkeys, pigs, dogs, sheep, calves) (8). In the dog, the LD_50/30_ is between 2.55 and 2.81 Gy (18, 20). Still, these values are of limited interest for the scope of the study as they highly overcome potential radiation thresholds during pregnancy in dogs.

### 1.4 Use of diagnostic imaging in the pregnant bitch

In veterinary medicine, as in human medicine, RI was the earliest available technique for evaluation of internal structures. With the discovery of UE a shift was observed, conditioning the modern use of X-rays mainly to the evaluation of skeletal structures and thoracic organs (10). UE instead allows a more detailed evaluation of abdominal organs and their structural integrity (10). Furthermore, it must be considered that RI provides a two-dimensional static image, whereas UE allows a dynamic exam allowing also the visualization of blood flow (Doppler ultrasound). Although, MR and CT-scans are performed in a routine manner also in veterinary medicine, ultrasound examination and X-ray remain the most frequently used techniques. This is mainly due to their availability, ease of use and lower costs, when compared to CT-scan and MR as well as the fact that anesthesia is required for the two latter.

In the pregnant bitch, the usefulness of both UE and X-ray is determined by the day of gestation. Ultrasonography allows pregnancy diagnosis as well as evaluation of viability of the pregnancy. In the canine species, hormonal tests for pregnancy diagnosis are available, yet ultrasonography remains the technique with the earliest reliable diagnosis, that also allows to assess embryonic viability, around the third week of pregnancy. RI allows the visualization of skeletal fetal structures during the last week of gestation. Both techniques have advantages and disadvantages, yet should be considered separate exams with different diagnostic values (24–32). UE, which may be used in all stages of pregnancy, allows the diagnosis of pathological processes early in the pregnancy and evaluation of the viability of embryos and later on fetuses. RI, in opposition, is used primarily for the determination of number of fetuses toward the end of pregnancy (29). Over the last two decades, RI has been proposed to have predictive value regarding the possibility of dystocia using pelvimetry and the comparison of fetal head size and diameter of the pelvic canal (28, 30, 33). X-rays in the pregnant bitch are performed either as a single latero-lateral image or a radiographic study consisting of two images in latero-lateral recumbency (right and left) and one in ventro-dorsal recumbency to allow measurements for pelvimetry (28, 33). UE instead may be performed with the bitch in standing position or in recumbency, although images obtained with the ventro-dorsal recumbency are preferred as it allows easy evaluation of both uterine horns.

### 1.5 Breeders perception and the role of the veterinarian

In recent years, a shift in breeders’ opinion regarding diagnostic imaging in the pregnant bitch was observed. RI in the last stages of gestation have been considered useful and were routinely performed over a long period of time. Nowadays, veterinary practitioners report that they are more frequently confronted with breeders doubts and skepticism regarding the usefulness and possible negative side effects of RI in pregnant bitches. The reasons behind the owners’ hesitation and sometimes, rejection, toward RI during the end of pregnancy comprise the stress caused to the bitch during the positioning, the dangers of radiation both for the bitch and for the fetuses and even the potential harm for the bitches’ and fetuses’ gonads. On the other hand, breeders recognize the importance of the X-ray at the presumed end of pregnancy that ensures the expulsion of all pups and the end of parturition; which UE cannot provide, especially in advanced stages of pregnancy. Breeders are keen to protecting the bitch from stress and recognize the importance of not separating the bitch from the pups in the first hours of life and also the dangers of moving the pups. Thus, it is controversial that breeders favor transporting the bitch to the veterinary for an X-ray after the presumed conclusion of the birthing process, to ensure all fetuses have been expelled, than performing it beforehand while the fetuses are still *in utero*. While stress may represent a valid concern for breeders, despite the lack of evidence regarding the impact of stress caused by a non-invasive examination during pregnancy, the fear of potential side effects on the bitch and fetuses caused by the X-ray is still nowadays unsubstantiated. Stress resulting from X-rays in the final stage of pregnancy may be assessed clinically and also managed by ensuring a calm environment for the bitch and reducing manipulations and the required time to the minimum necessary. Regarding the potential negative effects on the gonads, there are currently no diagnostic tools that allow this evaluation. Therefore, it is extremely challenging to determine the impact of radiation exposure of the few X-rays performed in end-stage pregnant bitches. General and reproductive disorders are multifactorial, being influenced by environmental and genetic factors. In the case of presence of genetic mutations, hypo- or infertility or neoplastic development either in the bitch or its descendants, it would be virtually impossible to trace them back to a radiographic study performed during pregnancy. Despite the absence of studies tackling this matter, it is important for general clinicians, reproductive specialists and researchers to understand breeders’ concerns and motivations in order to promote transparency and informed communication between the two parties but also to adapt and redirect research efforts. Communication is key between the owner (breeder) and the veterinarian. Yet, from the veterinarian’s perspective, some breeder’s concerns are scientifically unfounded, regarded as myths and promptly dismissed. This conduct may hinder the relation between owner and veterinarian and contribute to incompliance of the former. Clear communication, taking seriously breeder’s apprehensions helps to build a trust relationship, allowing for owner’s education and receptiveness to veterinarians’ suggestions. In this context of insufficient evidence, it is important that breeders are informed as such, yet giving them all the available information in order for them to make an informed choice and to ease their concerns when agreeing to a radiographic study in the end of pregnancy.

### 1.6 Stress and pregnancy

Stress is defined as a sense relating to adversity, strain or exertion due to either physical or mental factors (34). Stress can be categorized as acute stress when it results from a specific, temporary event like a visit to the veterinary doctor, an X-ray or ultrasound with physical restraint, or as chronic stress when it is the result of an ongoing situation like pregnancy or post-partum period. Although, stress and its effects on reproduction have been well studied in women, little is known on its effects on domestic animals (35). Studies concentrate mainly on laboratory animals such as mice and rats. Subjects are exposed to different types of stressors such as containment, food and water intake restrictions and noise (36). Negative correlation between stress and pregnancy rate, average litter size and normal corpora luteal formation have been found. Circadian rhythm also plays a role in the impact of stress on pregnancy - it has been shown that animals exposed to the stressor (containment) during the first part of the day (a.m.) suffered significantly more consequences than animals exposed later during the day (p.m.) (36). Effects of stress on early pregnancy in the pig were investigated in a prospective study by Einarsson et al. (37). Einarsson et al. define stress as a disturbance of homeostasis linked to enhanced activity of the hypothalamo-pituitary–adrenal (HPA) axis. To investigate effects on early pregnancy, pigs were exposed to different types of stressors such as changes in housing, food deprivation and temperature. All three stressors had a negative impact on conception rates, number of viable embryos, embryonic death, and average litter weight at birth and litter size (37). In 2019, a review was published by Nagel et al. (38) summarizing available literature on the effect of stress on parturition in domestic animals. The targeted animals were mainly cattle, horses, sheep and goats and the effect of transport right before or during parturition. Very little is known on the canine species. Cortisol was measured to determine the level of stress the animal was subjected to (38). A work from 2021 studied stress from mating until 60 days postpartum in the bitch by using a new noninvasive method of specimen collection. Nail trimmings and coat were collected to measure cortisol which is related to the activation of the HPA axis (39). Results showed that cortisol levels increased both in the nails and hair of bitches throughout pregnancy and the post-partum period reflecting the role of maternity on inducing chronic stress in bitches (39). Opinions and views of French dog breeders were collected using a questionnaire regarding different practices, observed behaviors and estimation of stress in their respective bitches (40). Breeders considered restlessness and excessive wandering as signs for significant stress during the immediate period before parturition and during parturition. Although trembling and panting are to be considered physiological behavior during parturition, owners have included these behaviors as signs of stress (40). In 2021 the same group of researchers made the questionnaire available to other countries to understand differences in breeding practices between different countries (41). Although agitation, pacing and restlessness were the most commonly named signs of stress, breeders also selected aggressiveness toward people, barking and whining (41). In other species, mainly rats and primates, high levels of maternal stress in the pre-natal period were associated with psychological and physiological effects in the offspring through epigenetic mechanisms (42, 43), contributing to abnormal or stereotypical behaviors (44), increased fear (45–47), overactive reactions to stressors (48), pre-term births (49), decreased birthweights (50), increased number of stillbirths (51) and compromised immune functions (52). Therefore, clinicians should pay special attention to minimizing stress in pregnant bitches during veterinary consults and necessary veterinary examinations like UE and RI. Some techniques that may help controlling stress levels during these procedures are maintaining positive human contact, especially by the owner when possible, using low-stress handling techniques resorting to blankets and positioning cushions during ultrasound to increase comfort, reducing the time of the examination to the minimum indispensable when the bitch is clearly under stress, and employing positive reinforcement techniques using food treats, petting, praise or toys (53).

## 2 Discussion

Exposure to radiation and its effects on health have been widely studied in the human, with an increase of attention following global political and economical changes. Health effects such as acute radiation sickness and cancer development are recognized and may be considered also public knowledge. This knowledge led to a certain distrust toward war and not war-related use of atomic energy. The use of radiation in medicine on the other hand is overall considered an advantage as it increases diagnostic accuracy and allows treatment options which would otherwise be unavailable. Regardless of its usefulness, radiation exposure in human medicine is reevaluated once the patient is pregnant. Negative effects on the embryo and fetus have been described in humans and include teratogenic and carcinogenic effects (1). Although well studied in humans, studies and experiments in veterinary medicine on the effect of radiation exposure during pregnancy are nowadays limited to laboratory animals. Early studies using specimen of horse semen did not show any significant negative effect on semen quality parameters nor on fertility, gestation and health of the offspring post-insemination of irradiated semen (22). In another study, oocytes from irradiated ovaries (X-ray radiation with an absorbed dose of 100 mGy) were fertilized *in vitro*. A reduction in the rate of blastocyst formation was observed in all groups of embryos resulting from the oocytes exposed to radiation (54). Little to nothing instead is known on the effects of radiation exposure during pregnancy in the canine species.

UE is the preferred method for pregnancy diagnosis in the pregnant bitch. Despite its utility for diagnosing the presence of embryos at a rather early stage of gestation, the possibility to evaluate viability of the embryos and the monitoring of growth and development, studies have shown that UE is the inferior method for determining the number of fetuses compared to RI (24–32). To determine litter size with greater accuracy, studies suggest performing two images in latero-lateral recumbency (one left and one right). In order to assess the rapport between the pelvic canal and the fetal head diameter, a third image in ventro-dorsal recumbency is needed. Findings regarding its utility in estimating risk of dystocia vary, which may be due to incorrect positioning, incorrect measurements or breed-specific particularities (28, 30, 33).

In veterinary medicine, to obtain high quality images the animal must be physically or pharmacologically restrained. Sedation and anesthesia are to be avoided, if possible, during pregnancy, as anesthetic drugs reach the fetuses via placental circulation (55, 56). Pharmacological restraint of a pregnant bitch in order to perform X-rays (33), exclusively for informative reasons rather than out of diagnostic necessities, has to be viewed critically. Physical restraint should be preferred, which in turn exposes the operator to radiation (10) while potentially inducing stress to the bitch (57).

The exposure to radiation resulting from a single X-ray or a radiographic study in a bitch during late-stage pregnancy is reputed to be negligible. At this stage, the organogenesis of the fetuses is complete (58). Yet, breeders tend to follow the conservative hypothesis that as long as there is no certainty of safety, the procedure remains dangerous. Studies report that a dose of <50 mGy does not interfere with pregnancy and does not influence its outcome in women (59). The stage in pregnancy in which embryos or fetuses are exposed to radiation influences the degree of hazard for the fetuses. Yet, reported thresholds in human medicine for permanent consequences for the fetuses range between 60 mGy and 1,000 mGy (59). A single radiographic image without contrast enhancement may generate a fetal radiation exposure between <0.001 rad (<0.01 mGy) and 0.62 rad (6.2 mGy), standing far beneath the threshold reported as potentially harmful for human fetuses (14). No studies have been published regarding dangerous exposure levels in domestic dogs.

Regardless of the absence of literature or studies confirming negative side effects on either the bitch or the fetuses, some authors mention that X-rays in the pregnant bitch should be performed with caution due to the negative health effects of ionizing radiation in the case of repeated exposure resulting from multiple images (24, 25, 29). Other studies instead, underline that organogenesis is completed at the moment of RI for litter size determination and therefore negative effects of ionizing radiation on organ development is very low (26, 60).

At different occasions and settings, the authors of the present study have been confronted with doubts of breeders, especially experienced ones regarding RI in their pregnant bitch. Different aspects and doubts have been named such as negative effects on the health of the fetuses, negative effects on the health of the dam and negative effects on physiological progression of gestation and parturition. No such doubts were raised regarding UE, which is in accordance with a study from Toal et al. (31). As studies are very clear about possible risks of ionizing radiation on different parts of the body, there is also an agreement in both human and veterinary medicine concerning the moment of exposure. The embryo and fetus are particularly sensitive to ionizing radiation during the first and second trimester of pregnancy when organogenesis is taking place (26). Regardless, breeders may be concerned both about the teratogenic as well as the carcinogenic potential of ionizing radiation. In the absence of recent studies in domestic animal species, veterinarian clinicians may use studies from human medicine as reference. These studies have shown that low doses of ionizing radiation (single radiographic images), did not cause a negative impact neither on the viability of pregnancy nor on the health of the neonate when compared to individuals exposed solely to background radiation (2, 14, 61, 62). These findings are supported also by studies in laboratory animals (23). Nevertheless, women as well as their physicians continue to be afraid of ionizing radiation during pregnancy which may lead to the decision to terminate a gestation in case of RI performed at early stages of pregnancy (14, 15). Furthermore, it has been described, that the ovaries of dogs have a comparatively higher resistance to radiation exposure (8). This has been shown by exposure to whole-body irradiation using sublethal doses which have failed to produce any consequences on reproduction both prior and after puberty (19).

Even though research is significantly more advanced in the human species when it comes to radiation exposure risks during pregnancy, caution should be taken when extrapolating the current knowledge into other species like the dog. Some thresholds available for women can also be employed in the bitches, for example 0.05 Gy as the maximum dosage below which no increased risk resulting from radiation exposure is expected to fall upon the pregnant women or the fetus. Even though, specific studies have not been performed to establish this threshold in bitches, it would be plausible that ionizing radiation dosages above 0.05 Gy may interfere with pregnancy also in the canine species. This hypothesis would lead to the recommendation that bitches should not be exposed to radiation dosages higher than 0.05 Gy during pregnancy, a value which is not even nearly reached with 2–3 X-ray projections for litter size estimation. However, it is also possible that this threshold may actually be different for the dog due to specific variations such as body mass, tocicity (monotocous for the human vs. polytocous for the dog) and duration of gestation (approximately 9 weeks in the dog and 9 months in the human), which also determines different durations for blastogenesis (2–17 days vs. up to 2 weeks), organogenesis (19–35 days vs. 2–7 weeks) and fetogenesis (35–61 days vs. 8–38 weeks), respectively in the dog and human (2, 63). Thus, the lack of research on threshold values for the canine species leads to the necessity of comparison with those given for humans. The established limit for humans should not be remotely reached as it may be inferior for the canine species due to the above mentioned differences in physiology and anatomy.

Teratogenesis and carcinogenesis are multifactorial processes which depend not only on the exposure of the embryo to external influences such as radiation or medication but are also influenced by genetic components or singular point mutations. When considering isolate cases, it is therefore unattainable to prove that certain malformations/negative health effects resulted from exposure to RI.

Breeders seem to be further concerned with the effects of stress on the pregnancy and parturition of their bitch. Stress has been linked to negative effects on several aspects of human reproduction, such as conception (64). Stress as well as pain are subjectively perceived, and its quantification is not standardized. In human medicine, researchers have different possibilities to determine stress in their patient, including measurement of stress-related hormones and changes in clinical parameters (e.g., cardiac frequency) (65). Most importantly human medicine doctors, differently from veterinarians, have the possibility to speak to their patients which is necessary to understand the perceived level of stress. Perceived level of stress by the animal may be described and tentatively assessed by the owner and veterinarian, which although capable of noticing eventual changes in behavior, represent nevertheless a third party. Owners perception of the animal’s stress depends on many different factors which leads to great difficulty in the evaluation of such data. Even behaviors considered as physiological during parturition have been described by breeders as a sign for increased levels of stress (40, 41). Techniques for measurement of cortisol as the main stress-related hormone have improved, giving researchers the possibility to collect specimen in a non-invasive manner (39). Collection of hair and nails are non-invasive techniques, but cortisol levels in these substrates do not reflect acute stress but chronic (weeks to months) stress. Furthermore, it is possible that especially nail trimming is perceived by the animal as a stressful manipulation. Visits to the veterinarian in general, unrelated to pregnancy and parturition, are frequently experienced as stressful by the animal (65). Perceived stress is also dependent on the character of the animal, prior experiences at the veterinary clinic and the frequency with which animals are subjected to this situation (65). Nagel et al. (38) reviewed the available literature on the effect of stress in the immediate peripartum time and consider animal transport prior or during parturition as an important stressor which may lead to complications during parturition (38). Especially transport in combination with the separation of the dam from the offspring are considered stressful (38).

The bitch’s positioning in ventro-dorsal recumbency for the radiographic study may lead to a significant amount of stress. Studies so far have assessed cortisol levels in the parturient animal and conclude that manipulation should be reduced to a minimum during the birthing process (38). However, no publications have yet established the effect of stressful events days before parturition. Although knowledge about this specific aspect is limited, it may be assumed that the bitch’s character and prior experiences with veterinarians play an important role on the level of stress experienced. Bitches which are very frightened or stressed when presented to the veterinarian prior to pregnancy, will not react differently in late-stage pregnancy (65).

Although experienced breeders may consider determination of number of fetuses as unnecessary, RI after the presumed end of parturition is a frequently used practice to determine that all pups have been expelled correctly. This particular practice may be difficult to understand for many clinicians, as bitches are exposed to an important stressor in a moment of great importance which may leave the clinician to question whether the stress of RI during the last week of gestation may be considered much lower than the stress of RI during parturition or immediately after, considering also the proven evidence of the negative effect of the separation of the dam from the newborn. Another consideration is the exposure of the dam to the environment of the veterinary clinic immediately post-partum or intra-partum. Hygiene is of great importance in any veterinary clinic, yet the load of viruses and bacteria present within such an environment is significant (66). Regardless of all safety precautions, bitches are exposed to these infectious agents in a vulnerable moment.

## 3 Conclusion

Up until today there is an important lack of data regarding the effects of exposure to low-dose ionizing radiation during DI in domestic animal species. Although, single radiographic images are considered safe in human medicine and of low risk in the pregnant woman, breeders have shown increasing concern regarding the use of RI in the pregnant bitch. Determination of teratogenic, carcinogenic or otherwise negative health effects on the fetus and the dam itself are difficult if not impossible to study, as those processes are multifactorial in their development. Stress has been proven to have a negative effect on reproduction but there is no data available on the amount of stress dams are exposed to during X-ray for puppy count. Studies are necessary to evaluate these stress levels prior to concluding that X-rays in late-stage pregnancy causes high levels of stress. Up until today, no studies are available which may support breeders’ concerns, yet at the same time there is little data available which may reassure breeders of the safety of X-rays in late-stage pregnancy.

The use of X-ray for litter size determination should therefore be discussed with the owner and evaluated on a case-to-case basis while also taking the character of the bitch into consideration in order to minimize stress during this particular moment. Until new evidence is available on the safety of RI during pregnancy, the authors’ recommendations are to limit X-ray use to late gestation (from 45d onwards), avoid unnecessary imaging, minimize maternal stress, prefer low-stress physical restraint over chemical restraint (sedation), communicate transparently with breeders acknowledging their concerns and emphasizing that the current evidence supports late-stage pregnancy radiography safety and respect breeder’s input tailoring decisions to each individual case, involving the breeder in risk–benefit discussions.
